# Supplementary material for: Early Effects of Extracellular Vesicles Secreted by Adipose Tissue Mesenchymal Cells in Renal Ischemia Followed by Reperfusion: Mechanisms Rely on a Decrease in Mitochondrial Anion Superoxide Production
Source: Int J Mol Sci. 2022 Mar 8;23(6):2906. doi: 10.3390/ijms23062906 (PMC8955255; doi:10.3390/ijms23062906)
Supplement: Supplementary file 1 [file ijms-23-02906-s001.zip › ijms-1602176-supplementary.pdf]

International Journal of
Molecular Sciences

MDPI

Supplementary Material

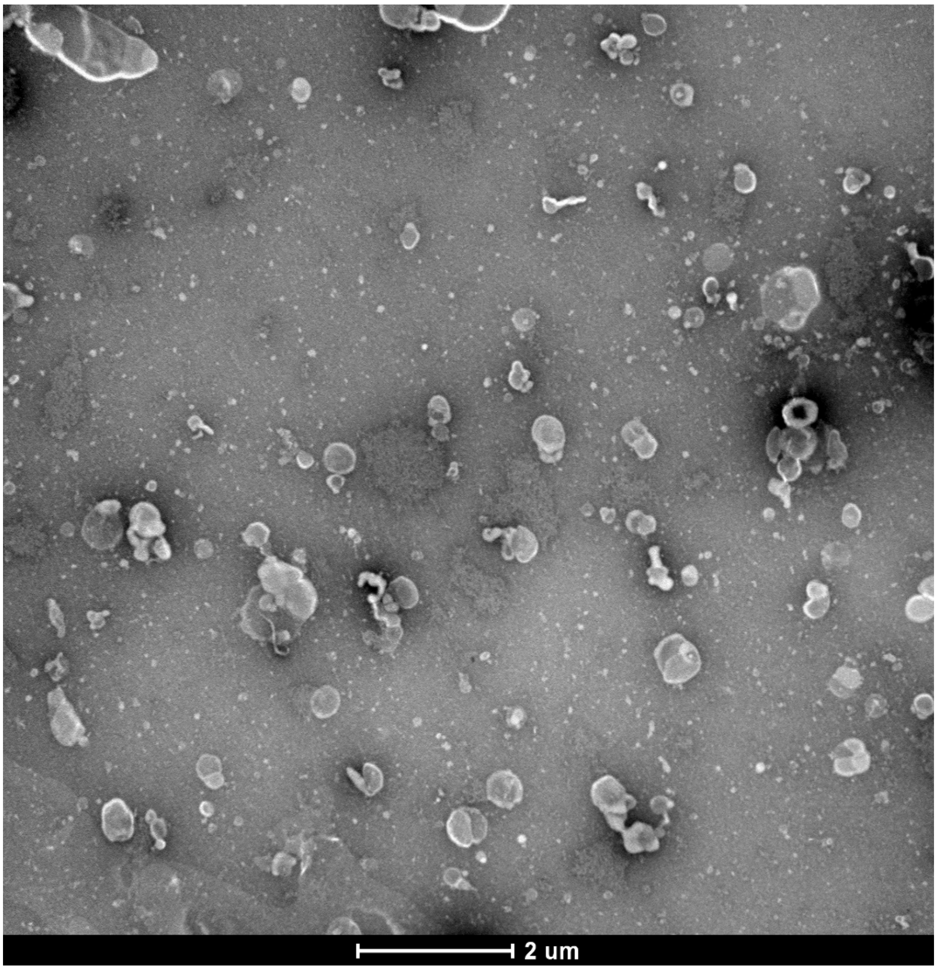

Figure S1. A. Raw image of Figure 1A (left) from the main text.

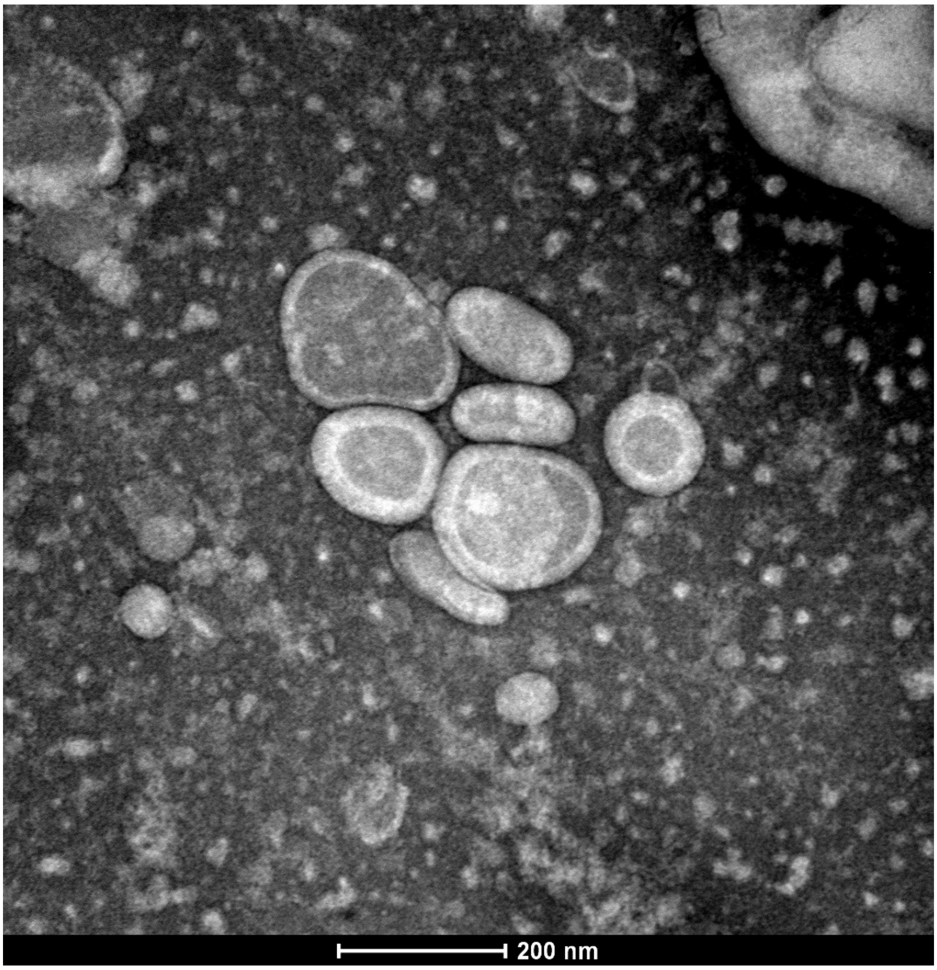

Figure S1. B. Raw image of Figure 1A (right) from the main text.

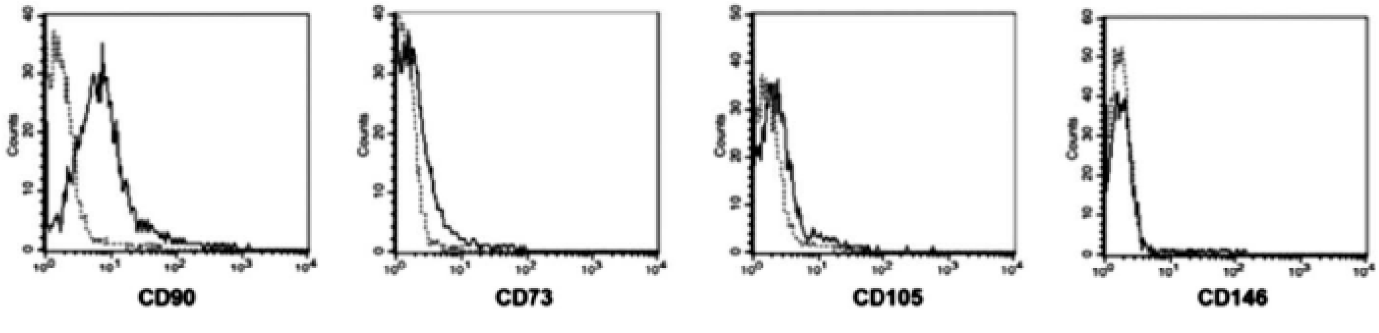

Figure S2. CD90, and CD105, but not CD146, in EVs isolated from cultures of adipose MSC. The dotted traces at the left of the panels correspond to EVs only incubated with secondary IgG as a negative control. Partial reproduction of Supplementary Figure 2 from [10] under the Creative Commons License Options.

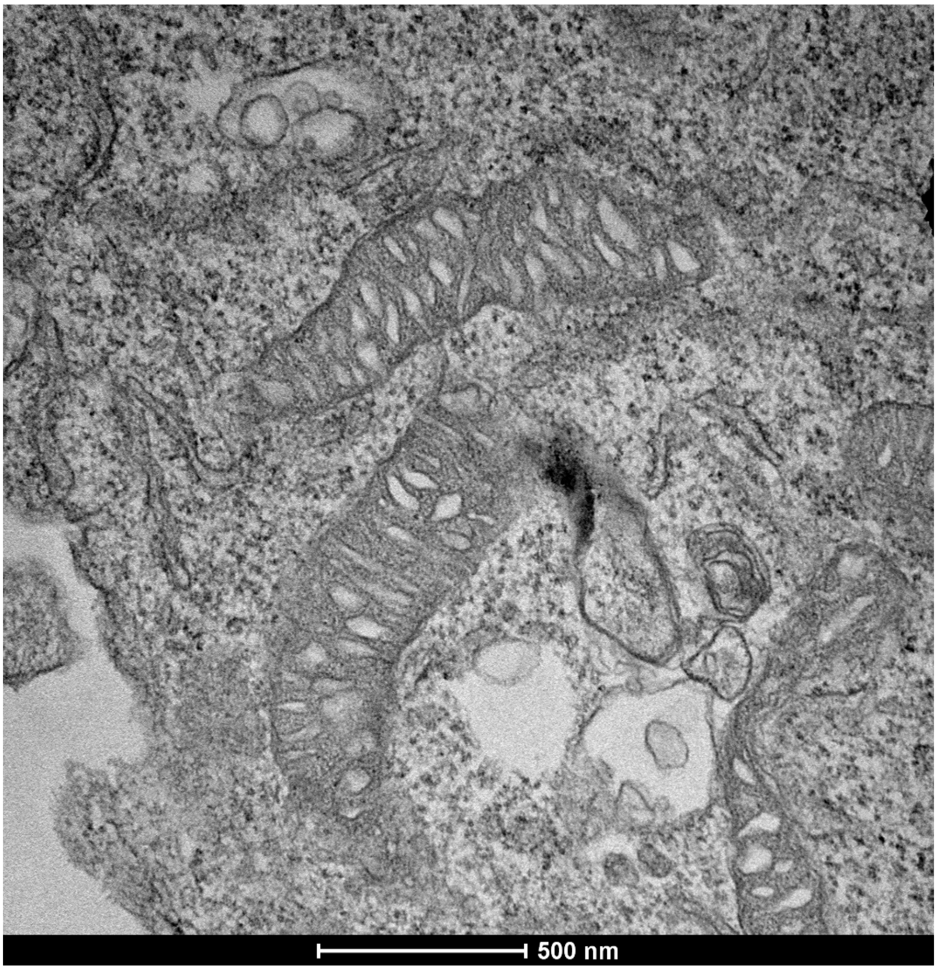

Figure S3. A. Raw image of Figure 3A (CTR) from the main text.

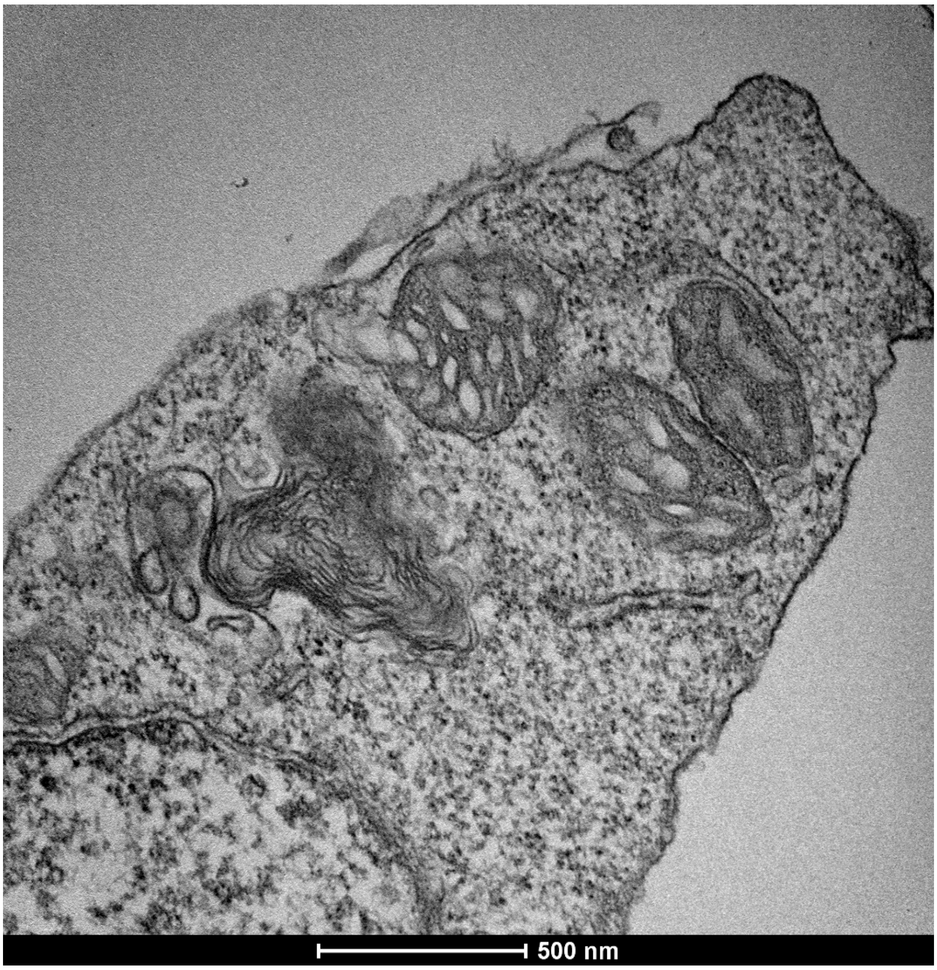

Figure S3. B. Raw image of Figure 3A (HPX) from the main text.

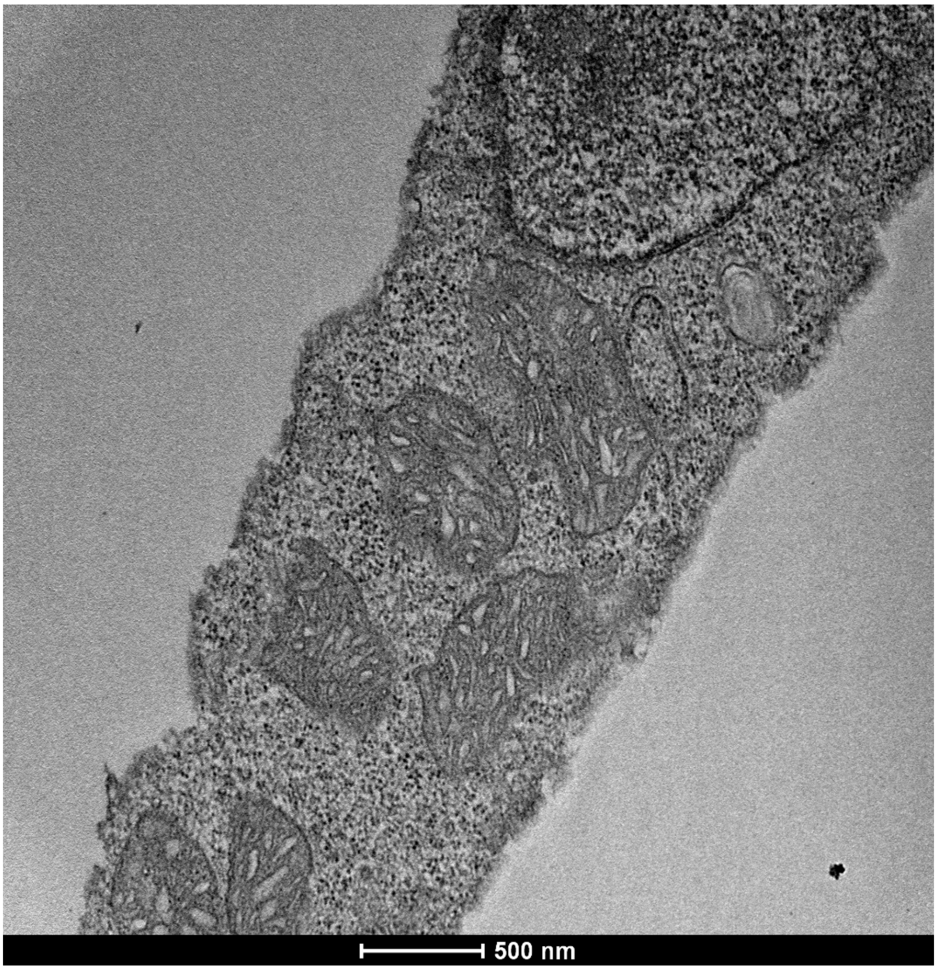

Figure S3. C. Raw image of Figure 3A (HPX+EVs) from the main text.
